# Bayesian Model for Matching the Radiometric Measurements of Aerospace and Field Ocean Color Sensors

**DOI:** 10.3390/s100807561

**Published:** 2010-08-11

**Authors:** Mhd. Suhyb Salama, Zhongbo Su

**Affiliations:** Faculty of Geo-Information Science and Earth Observation (ITC), University of Twente, Hengelosestraat 99, 7500 AA Enschede, The Netherlands; E-Mail: b_su@itc.nl

**Keywords:** Bayesian, maximum entropy, match-up, ocean color, MERIS

## Abstract

A Bayesian model is developed to match aerospace ocean color observation to field measurements and derive the spatial variability of match-up sites. The performance of the model is tested against populations of synthesized spectra and full and reduced resolutions of MERIS data. The model derived the scale difference between synthesized satellite pixel and point measurements with R^2^ > 0.88 and relative error < 21% in the spectral range from 400 nm to 695 nm. The sub-pixel variabilities of reduced resolution MERIS image are derived with less than 12% of relative errors in heterogeneous region. The method is generic and applicable to different sensors.

## 1. Introduction

Consistent and accurate matching of aerospace observation data to field measurements are necessary calibration and validation steps towards creating reliable products of inherent optical properties (IOPs). The scientific procedure to match satellite observations to field measurements can generally be divided into three steps: *i-* measure remote sensing reflectance above the target in an area with homogenous optical properties; *ii-* re-sample the pixels of satellite data that surround the field site; *iii-* match the values obtained from step *i* to those computed from step *ii*. Accurate estimation of the sub-scale variability of the match-up site and its inclusion in the matching method is the most suitable approach to calibrate earth observation retrieval algorithms and validate their products.

Matching procedures for ocean color sensors were addressed by many researchers. For instance, Harding *et al.* [1] selected field measurement sites in homogenous areas and Bailey and Werdell, [2] averaged a number of spatially-homogeneous pixels surrounding the match-up site. Although, aggregating ocean color pixels was found to be suitable for open ocean [3,4], it lowers the percentage of usable match-up points considerably and should be avoided in coastal waters [5]. Any direct matching in coastal turbid waters may result in large discrepancy [6,7]. Hyde *et al.* [8] recognized that the mismatch between field measurement and SeaWiFS products of chlorophyll-a is partially due to difference in the sampling scales and therefore introduced a correction factor to overcome this scale mismatch.

In this paper we introduce a complete scheme to quantify the scale difference between a satellite pixel and a point (field) measurement. We used Bayesian inference method [9] to estimate the probability distribution function (PDF) of the match-up pixel using the deviations between field and satellite measurements. We will discuss the methodology and performance of the model as applied to radiative transfer simulations [10, IOCCG data set] and MERIS images at full and reduced resolutions acquired over the Dutch Bight.

## 2. Method

### 2.1. Ocean color model

In this study we will use the model of Gordon *et al.* [11] to relate the observed remote sensing reflectance that is leaving the water body *Rs_w_*(*λ*) to the water biophysical properties:

(1) $${\mathrm{Rs}}_{w}(\lambda )=\frac{t}{n_{w}^{2}}\sum_{i=1}^{2}g_{i}{\left(\frac{b_{b}(\lambda )}{b_{b}(\lambda )+a(\lambda )}\right)}^{i}$$

where *g*_1_, *g*_2_ are subsurface expansion coefficients due to internal refraction, reflection and sun zenith; *t* and *n_w_* are the sea air transmission and water index of refraction, respectively. Their values are taken to be: *g*_1_ = 0.0949, *g*_2_ = 0.0794, *t* = 0.95, *n_w_* = 1.34. The quantities *b_b_*(*λ*) and *a*(*λ*) are the bulk backscattering and absorption coefficients of the water column. Case II water is considered with five independently varying constituents, namely: water molecules, chlorophyll-a (Chl-a), detritus, dissolved organic matter and suspended particulate matter (SPM). The absorption *a*(*λ*) and backscattering *b_b_*(*λ*) coefficients are parameterized as reported in Salama *et al.*[12,13] and summarized in Appendix 1. We will consider three main variables as derived from (1) using the method of Salama *et al.* [12]. These variables are: (i) – the absorption coefficient of Chl-a at 440 nm, *a_phy_*(440); (ii) – the combined absorptions of detritus and dissolved organic matter at 440 nm, *a_dg_*(440); (iii) – the scattering coefficient of SPM at 550 nm, *b_spm_*(550). The derived variables are called the set of IOPs and denoted in a vector notation **iop**.

### 2.2. Bayesian inference

Our basic assumption is that each pixel in aerospace ocean color data represents the mean of an unknown theoretical probability distribution function (PDF) over a pixel area. In this respect, any field measured radiance is a sample drawn from this PDF [14]. Our objective is to estimate the standard deviation of this PDF which represents the scale difference between pixel and point measurement. This standard deviation is also equal to the sub-scale variability of the satellite pixel. We will use the words (sub-scale variability) to represents the scale-difference and quantify the standard deviation as its measure.

The radiometric mismatch, between aerospace and field sensors, is attributed to the scale difference and is represented as an upper and lower bounds with a 1−*α* of confidence using the method of Bates and Watts [15]:

(2) $${\mathrm{Rs}}_{\pm }=\mathrm{Rs}(\lambda )\pm \sigma \left\|\frac{\Delta \mathrm{Rs}(\lambda )}{\Delta \text{iop}}R^{-1}\right\|t(N-m,\alpha /2)$$

where, *Rs*_±_ is the upper “+” and lower “−” bounds of *Rs*(*λ*); *Rs*(*λ*) is the observed radiometric quantity; *σ* is the standard deviation of the radiometric difference between field and aerospace measurements; *t*(*N* − *m*, *α*/2) is the upper quantile for a Student’s *t* distribution with *N* − *m* degrees of freedom. *N* is the number of bands and *m* is the number of unknowns. **R** is the upper triangle matrix of QR decomposition of the jacobian matrix. The derivative term in (2), can be approximated as being the gradient of (1) with respect to the derived IOPs and is computed for model-best-fit to the observation. This approximation is derived in Appendix 2.

The plausible range of the IOPs can be estimated from the upper and lower radiometric bounds by simply inverting (1) for the upper and lower radiometric bounds. A first estimate of the IOPs standard deviation is then derived form their plausible range using the method of Salama and Stein [9]. This method is summarized in Appendix 3. Campbell [16] showed that marine biophysical quantities are most likely log-normally distributed. We, therefore, use a log-normal distribution to generates random IOPs values within their plausible range using the estimated variance. We call these generated IOPs values a prior PDF of IOPs. The posterior probability of IOPs is then derived by maximizing the cross entropy H between prior and posterior information [17,18]:

(3) $$H=\sum_{1}^{N}P(\text{iop}|\ell )\cdot \text{log}\frac{P(\text{iop}|\ell )}{P(\text{iop})}$$

where P(**iop**) is the prior probability and P(**iop**|*ℓ*) is the posterior probability of **iop** given the IOPs range *ℓ.* The empirical PDF of spectra is estimated from permutated values of the derived posterior PDF of IOPs. The importance of permutation is to simulate the ambiguity of remote sensing reflectance [19] with respect to different sets of IOPs, *i.e.*, similar spectra corresponding to different sets of IOPs.

### 2.3. Algorithm

The above-mentioned theoretical derivation of the Bayesian inference are summarize in the following algorithm:

1. use (2) to estimate the upper and lower bounds of spectra;
2. derive the IOPs ranges from these spectra by inverting (1);
3. use the method of section 3 to estimate the standard deviation of IOPs from their range;
4. generate the prior PDF of IOPs using log-normal distribution;
5. initiate the posterior using log-normal distribution;
6. maximize (3) to obtain an new estimate of the posterior;
7. update the posterior;
8. iterate between steps (5) and (6) till convergence;
9. permutate the resulting PDF of IOPs;
10. forward the sets of IOPs using (1) to obtain the empirical PDF of spectra.

## 3. Results

### 3.1. Simulated data set

**Figure 1.**
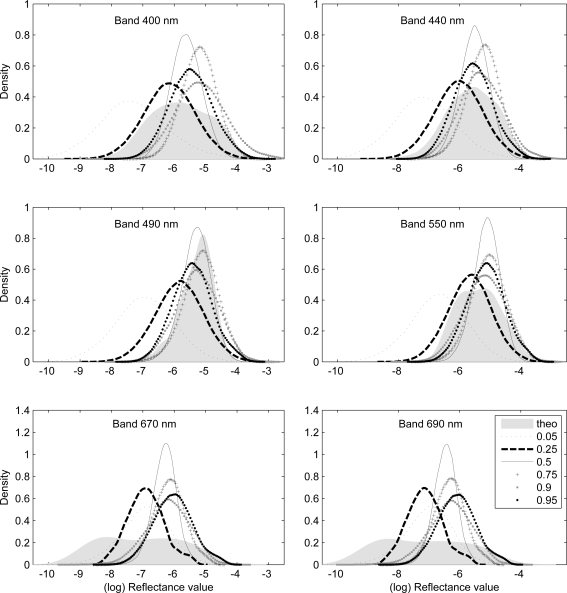
Empirical PDFs generated from field spectra corresponding to predefined quantiles: 0.05 “doted line”, 0.25 “dashed line”, 0.5 “full line”, 0.75 “plus”, 0.9 “square”, 0.95 “circles” for six wavelengths. The theoretical PDF is illustrate as gray area.

The proposed model was applied on [10, IOCCG data set] of radiative transfer simulations at 30° sun zenith for synthesized sets of IOPs. The spectral arithmetic mean of this data set was computed to represent the observed aerospace spectrum. Spectra that corresponded to quantiles 0.05, 0.25, 0.5, 0.75, 0.9 and 0.95 were chosen to represent probable *in-situ* observations. The empirical distributions of the underlying population were derived and plotted in Figure 1.

Note that reflectance values are also log-normally distributed. Figure 1 shows that the distribution resulting from the 0.5 quantile has the largest kurtosis and the smallest deviation from the theoretical mean. We performed modified Ansari-Bradley test [20] at 5% significant level on the PDFs in Figure 1. This technique is a non-parametric two-samples test on dispersions of the theoretical and empirical PDFs. The results of the Ansari-Bradley test are shown in Table 1 as probabilities of the empirical PDF having the same dispersion as the theoretical PDF for radiometric and geo-biophysical quantities.

**Figure 2.**
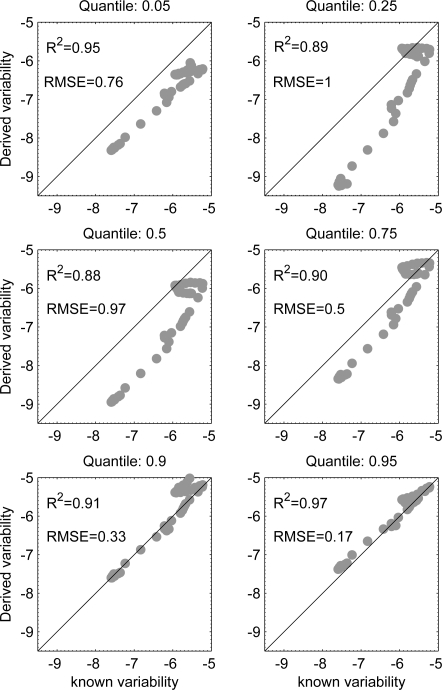
Known *versus* derived variability corresponding to predefined quantiles on a log-scale of radiometric data. The 1:1 line is also shown.

The Ansari-Bradley test shows that wavelength 495 nm is more probable to produce the sought variability regardless of the position of *in-situ* measurements in the theoretical PDF. Our model is more likely to derive the variability of all IOPs from the 0.95 quantile. The probability of deriving the variability of *a_dg_*(440) and *b_spm_*(550) is higher than deriving the variability of *a_phy_*(440). Figure 2 shows the relationships between the known and derived standard deviation with root-mean of squared errors (RMSE) and R^2^ values derived from model-II regression [21] of log-transformed radiometric data. The variability obtained from all quantiles have very close values to the theoretical variability with R^2^ larger than 0.88. The RMSE values almost increase towards the median and start decreasing to reach a minimum value of 0.17 for 0.95 quantile. Close quantiles to the median (0.5) reproduce PDF with small dispersions. Table 2 shows the relative errors between known and derived (log) standard deviation. For all combinations of quantiles and wavelengths, the retrieved variabilities of reflectance were within 21% of known values. The root-mean of squared relative errors (RMS-RE) between known *c* and derived *x* values was computed for all wavelengths as shown in the last row of Table 2:

(4) $$\text{RMS}-\text{RE}=\sqrt{\frac{1}{n}\sum_{i=1}^{n}{\left(100\frac{x_{i}-c}{c}\right)}^{2}}$$

where *n* in this case is the number of bands. The best results are obtained from field spectrum corresponding to the 0.95 quantile and RMS-RE values have the same trends as RMSE values shown in Figure 2, *i.e.*, increases towards the median from both sides.

### 3.2. Model stability to atmospheric and random noise

**Figure 4.**
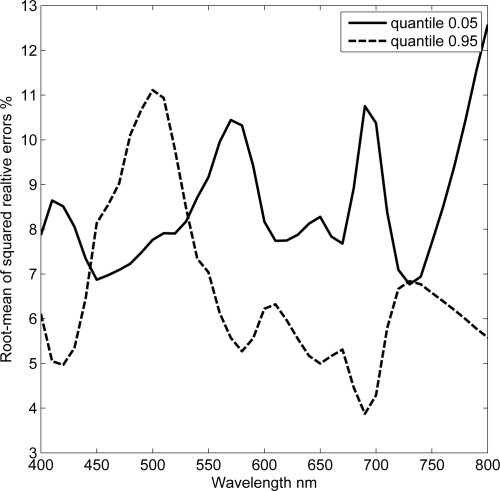
RMS-RE values in estimated *σ* as function of wavelength. The values of *σ* were derived from fluctuated mean and two field spectra corresponding to 0.05 and 0.95 quantiles.

The stability of the model to uncertainties in atmospheric correction and sensor noise was tested by perturbing the mean, *i.e.*, aerospace spectrum, with random normal fluctuations. The random perturbations were assumed to be wavelength dependent with zero mean and standard deviation calculated from the theoretical PDF. This implies that the residuals between the aerospace and field spectra are now due to three components, namely: errors originated from atmospheric correction, sensor noise and the spatial scale difference. The added fluctuations are within ±50% of the actual values as shown in appendix Figure 3. RMS-RE is computed between the log of known and derived estimates using (4) with *n* being the number of fluctuations. Figure 4 shows that the RMS-RE values are less than 15% for derived variability values from the 0.05 and 0.95 quantiles. Derived values from 0.95 quantiles were more accurate than those derived using the 0.05 quantiles for wavelengths > 500 nm.

### 3.3. Ocean color radiometric products

**Figure 5.**
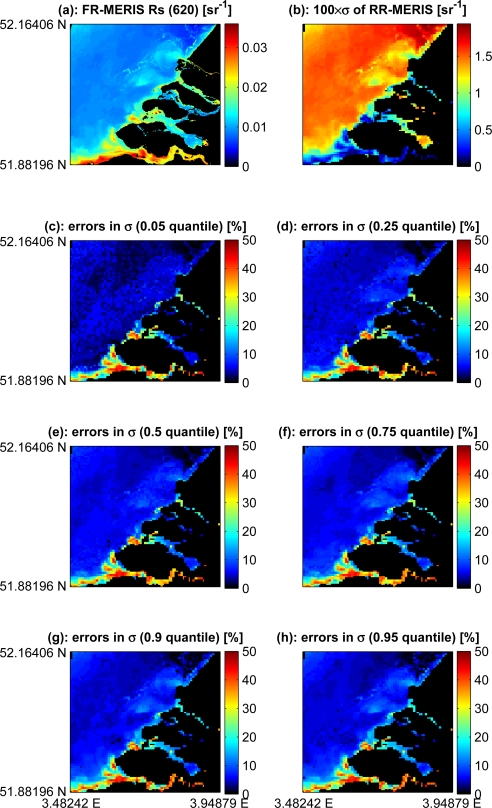
**(a)** Remote sensing reflectance of MERIS full resolution (FR) band 6 centered at 620 nm; **(b)** derived standard deviation of the reduced resolution (RR) MERIS image using the FR pixels; **(c–h)** relative errors of derived (log) standard deviation values of RR-MERIS corresponding to quantiles 0.05, 0.25, 0.5, 0.75, 0.9 and 0.95.

We used full (FR) and reduced (RR) resolutions of MERIS images acquired over the North Sea [22]. Atmospheric path correction was then performed using radiative transfer computation [23] and the method of Salama and Shen [24]. For this study, a region of the Dutch coastal waters was selected with relatively high optical variability. The sub-pixel variability of the reduced resolution image was derived for selected quantiles from the full resolution image. Figure 5 shows the relative errors of the derived variability for band 6 of MERIS centered at 620 nm. The derived (log) standard deviation values were within 10% of relative errors in spatially variable areas and up to 40% in spatially homogenous waters. The mean and standard deviation of RMS-RE values of the images in Figure 5 (c–h) were between 7% and 8% and 7.3% and 7.5% respectively.

## 4. Discussion

Our method is formulated based on two steps. First we estimate the plausible range of IOPs. Second, we derive the posterior PDF of IOPs. In the first step we use the method of Bates and Watts [15] to construct a bound-like around field and aerospace spectra. These spectra are inverted to derive the plausible range of IOPs. In the second step, we use the cross-entropy (3) as a utility function. The cross-entropy in (3) can be rewritten as: joint entropy minus the posterior’s entropy. Therefore maximizing (3) means maximizing the joint entropy between prior and posterior and minimizing the uncertainty about the posterior [18], resulting in an optimum posterior and maximum joint entropy. Equation (1) is then applied using the posterior PDF of IOPs to derive the empirical PDF of reflectance.

Validation with IOCCG data set shows that the root-mean of squared relative error is less than 15% for all possible field measurements. Moreover, derived values of variability are linearly related to the known values on a log scale with R^2^ > 0.88. Derived variability values from the green band, centered at ∼ 495 nm, are more probable and are invariant to the position of *in-situ* measurements in the theoretical PDF. The stability of the model is tested by imposing random fluctuations to the observed aerospace mean. The retrieved spatial variabilities from fluctuated data are within ±15% of the known values, with derived values from the 0.95 quantile being more accurate than those derived from the 0.05 quantile.

The proposed model is further tested with full and reduced resolution MERIS products covering part of the Dutch coastal waters. The highest errors in derived values of sub-pixels variability are in spatially homogenous areas. In these areas all quantile values are close to the mean and thus little information can be derived. This can also be observed in Figure 2. The results are slightly similar with respect to the different quantiles. However, derived variability from the 0.05 quantile was overestimated in turbid areas and provided good estimates in clear areas. The opposite was true for derived values using the 0.95 quantile, *i.e.*, better results were obtained in turbid waters. This is logic because in turbid waters it is difficult to find clear water pixels and in clear waters is difficult to find turbid water pixels. However, on the borders of the turbidity zone the method worked quite well. These areas exhibit the highest spatial variability at a given time. Our method derived accurate variability estimates in these edge-like areas. This behavior is apparent in Figure 5. Note the diagonal stripe from South-West to North-East separating the Dutch coastal waters and the high reflectance area in the North-West region of the image. This stripe indicates low error in derived variability.

Since atmospheric correction is a significant issue in water remote sensing [25], imperfect atmospheric correction can lead to a significant error if not properly treated. A proper treatment is to combine our Bayesian model with the method of Salama and Stein [9] to decompose the difference between satellite and field observations into error and scale components. The error component can be treated as in Salama and Stein [9] and the scale component can be quantified using our Bayesian model.

## 5. Conclusions

In this paper we developed and applied a Bayesian approach to address the scale variability between point and aerospace measurements above water. The model used the differences between the field and aerospace observed spectra to derive prior information on the IOPs. We then applied Bayesian inference to derive the optimum posterior distribution of IOPs by maximizing the joint entropy of the prior-posterior. Our approach provided information about the sub-scale variability of match-up pixel on the IOPs and radiometric levels. We, further, showed that match-up sites for radiometric quantity could be inhomogeneous and preferably located on the edge of the turbidity zone. Information on the sub-scale variability of geo-biophysical processes will facilitate planning of calibration and validation of future sensors, resolving the critical scale of variability of an observed feature and improving the assimilation of EO products into model grid and field data. Although the approach was developed for radiometric quantities in a match-up pixel, it has the potential to be applied on bio-geophysical properties using prior knowledge on their plausible ranges. In addition, we believe that our methodology is general and applicable to land surface studies. The same principle applies: utilizing prior knowledge about geo-biophysical quantities to derive sub-scale variability of satellite pixel. However, the proposed model needs a more extensive validation with different data sets on land parameters.

## Figures and Tables

**Figure 3.**
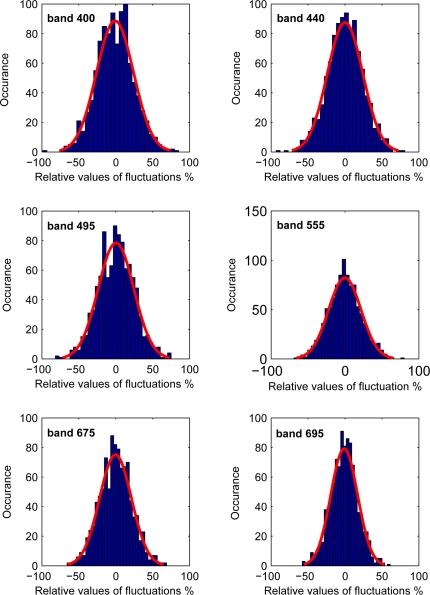
Relative values of fluctuations added to the aerospace mean for six wavelengths.

**Table 1.** Probability of empirical PDF having the same variability of the theoretical PDF.

	Quantile values					
band nm/IOPs	0.05	0.25	0.5	0.75	0.9	0.95
440	0	0.26	0	0	0.31	0
495	0.59	0.19	0.17	0.2	0.11	0.13
550	0	0.01	0	0.03	0.06	0.57

*a_phy_*(440)	0	0	0.09	0.17	0	0.18
*a_dg_*(440)	0	0.93	0.09	0.06	0.37	0.58
*b_spm_*(550)	0.33	0.21	0.34	0.51	0.05	0.3

**Table 2.** Relative errors (%) in derived log *σ* values of reflectance estimated from different quantile values. Minus values are underestimated. RMS-RE is the root-mean of squared relative error for all bands.

	Quantile values					
band nm	0.05	0.25	0.5	0.75	0.9	0.95
400	9.08	6.24	10.39	0.77	−13.12	−1.29
440	9.78	1.67	6.71	−1.65	−9.29	−1.16
495	7.29	−3.91	0.45	−7.32	−9.34	−5.74
550	15.35	4.57	8	−1.52	−3.06	−1.53
675	10.05	14.81	16.11	6.4	0.61	−0.4
695	13.77	20.7	20.99	11.12	3.84	2.32

RMS-RE	12.5	14.6	14.8	7.6	5.7	2.7

## Appendix

### 1. Parametrization of IOPs

The absorption and scattering coefficients of water molecules, *a_w_* and *b_w_*, were assumed constants and obtained from [26,27], respectively. The total absorption of phytoplankton pigments *a_phy_* is approximated as [28]:

(5) $$a_{\mathrm{phy}}(\lambda )=a_{0}(\lambda )a_{\mathrm{phy}}(0.44)+a_{1}(\lambda )a_{\mathrm{phy}}(0.44)\;\text{ln}\;a_{\mathrm{phy}}(0.44)$$

where *a*_0_(λ) and *a*_1_(λ) are empirical coefficients. The absorption effects of detritus and dissolved organic matter are combined due to the similar spectral signature [29] and approximated using the model [30]:

(6) $$a_{\mathrm{dg}}(\lambda )=a_{\mathrm{dg}}(440)\;\text{exp}[-s(\lambda -440)]$$

where *s* is the spectral exponent with value ∼ 0.021 nm^−1^. The scattering coefficient of SPM *b_spm_* is parameterized as [31]:

(7) $$b_{\mathrm{spm}}(\lambda )=b_{\mathrm{spm}}(550){\left(\frac{550}{\lambda }\right)}^{y}$$

where *y* is the spectral shape parameter ∼ 1.7. The backscattering fraction of SPM is estimated from the ”San Diego harbor” scattering phase function of Petzold [32], *i.e.*, *b_b_* = 0.0182 × *b*.

### 2. Estimating the gradient of radiometric observation

Observed remote sensing reflectance can be approximated as being the sum of the model best-fit *Rs_m_*(λ) and its deviations from the observed one ε(λ):

(8) $$\mathrm{Rs}(\lambda )={\mathrm{Rs}}_{m}(\lambda )+ɛ(\lambda )$$

The term *Rs_m_*(λ) is obtained from fitting the model in (1) to the radiometric observation of ocean color or/and field sensors. The error ε(λ) is a lumped term that includes model goodness-of-fit, measurements and atmospheric noises. For simplicity this term is assumed to be nearly independent the derived IOPs. The derivative of (8), with respect to the derived values, can then be written as:

(9) $$\frac{\Delta \mathrm{Rs}(\lambda )}{\Delta \text{iop}}=\frac{\Delta {\mathrm{Rs}}_{m}(\lambda )}{\Delta \text{iop}}+\frac{\Delta ɛ(\lambda )}{\Delta \text{iop}}$$

By definition of the least square minimization that was used to derive model-best-fit *Rs_m_*(λ), we have:

(10) $$\frac{\Delta ɛ(\lambda )}{\Delta \text{iop}}\approx 0$$

Equation (9) can then be reduced to:

(11) $$\frac{\Delta \mathrm{Rs}(\lambda )}{\Delta \text{iop}}\approx \frac{\Delta {\mathrm{Rs}}_{m}(\lambda )}{\Delta \text{iop}}$$

The simplification in (11) implies that the gradient of measured remote sensing reflectance can be approximated by the gradient of the model in (1) which can easily be computed.

### 3. Driving the standard deviation from the plausible range

Salama and Stein [9] has developed a method to estimate the standard deviation of a log-normally distributed population from its plausible range. Their method starts by generating random values from a normal distribution with zero mean and unity standard deviation, N(0,1). The generated values of N(0,1) satisfy an imposed acceptance-rejection condition. This condition requires that the ratio (13) defines a unique ordered pair of *α*:

(12) $$\alpha_{u}=\frac{{\text{iop}}_{u}-{\text{iop}}_{\mathrm{obs}}}{\sigma }$$

where **iop***_u_*, **iop***_obs_* and *σ* are the upper bound of the IOPs; the expectation (derived from model best-fit to observation) and the unknown standard deviation of the population, respectively. All IOPs are log-transformed first. For the lower bound of IOPs, **iop***_l_*, one can establish the ratio:

(13) $$r_{u,l}=\frac{\alpha_{u}}{\alpha_{l}}=\frac{{\text{iop}}_{u}-{\text{iop}}_{o}}{{\text{iop}}_{l}-{\text{iop}}_{o}}$$

Three look-up tables (LUTs) are then created from the generated N(0,1) values. These LUTs correspond to the following three scenarios:

(14) $$\begin{array}{ccccc} {\text{iop}}_{o} & > & {\text{iop}}_{u} & > & {\text{iop}}_{l}\\ {\text{iop}}_{o} & < & {\text{iop}}_{l} & < & {\text{iop}}_{u}\\ {\text{iop}}_{l} & < & {\text{iop}}_{o} & < & {\text{iop}}_{u} \end{array}$$

The ratio in (13) is first estimated from the log-transformed IOPs values. Based on the value of this ratio a lookup table is selected and searched to find the best-fit. One of the corresponding pair is then used in (12) to compute the standard deviation of the prior PDF.

### 4. Added random noise

Random normal fluctuations are assumed to be wavelengths dependent with zero mean and standard deviation calculated from the theoretical PDF, see Figure 3.
